# Synchronous Renal Malignancy Presenting as Recurrent Urinary Tract Infections

**DOI:** 10.1155/2011/832673

**Published:** 2011-09-18

**Authors:** G. Dutta, D. Silver, A. Oliff, A. Harrison

**Affiliations:** ^1^Department of Internal Medicine, Lutheran Medical Center, Brooklyn, NY 11220, USA; ^2^Department of Urology, Lutheran Medical Center, Brooklyn, NY 11220, USA; ^3^Department of Pathology, Lutheran Medical Center, Brooklyn, NY 11220, USA; ^4^Christian Medical College, Ludhiana, India

## Abstract

Renal cell carcinoma (RCC) and urothelial carcinoma of the upper urinary tract are not uncommon urological malignancies. Their simultaneous occurrence in a patient is, however, extraordinarily rare. We report the case of a patient who underwent unilateral nephrectomy for suspected RCC and diagnosed transitional cell carcinoma of the superior pelvis. Preoperative imaging was suspicious for renal pelvic involvement, which was confirmed upon performing cystoscopy and biopsy of the suspected lesion preoperatively. This preoperative approach was especially appropriate as a nephron saving procedure was being considered prior to the discovery of the synchronous lesion. We discuss this rare simultaneous occurrence of synchronous malignancies in the same kidney.

## 1. Introduction

Renal cell carcinoma (RCC) is the most common form, accounts for approximately 85%, of all kidney cancers and its incidence has been rising steadily in the last years. The concomitant association with urothelial carcinoma (UC) of the upper urinary tract in a patient is extraordinarily rare. The management for each of those, taken individually, could be significantly different. This becomes even more complicated when both lesions present simultaneously. Pre- or intraoperative recognition is important so that nephroureterectomy is performed. We report a case of synchronous RCC and UC of the ipsilateral renal unit.

## 2. Case Report

This is a case of a 71-year-old hispanic lady with past medical history of DM, HTN, dementia, and hypothyroidism, who presented to her PMD with c/o recurrent urinary tract infections, without any h/o gross hematuria, weight loss, or abdominal distension. A sonogram abdomen was done which showed an exophytic lesion at the inferior pole of the right kidney. For further evaluation, she was referred to the urologist. She denied having any known allergies. She gave history of smoking for many years and that she has quit smoking for few years now. No relevant significant family history was provided. To follow up on the sonogram findings, and as a preoperative workup, a CT scan abdomen/pelvis with renal mass protocol was obtained. It revealed an exophytic enhancing mass on the anterolateral aspect of lower pole, as seen already on the sonogram, in addition, an enhancing filling defect in the upper pole calyx with mild upper pole calyceal dilatation in the right kidney was also found. This second finding was suspicious for a second lesion in the same kidney. To correctly evaluate the nature of this second lesion, a cystoscopy with right ureteroscopy and biopsy were performed. During the procedure, a 1 cm papillary tumor was noted on entering the right upper pole calyx, which was biopsied. The biopsy result showed papillary transitional cell carcinoma. Interestingly, urine cytology (20 cc specimen) was found to be negative for malignant cells. 

With the revelation of this second primary tumor, a definitive procedure (right nephrectomy) was performed. Right kidney nephrectomy specimen showed clear cell type RCC with no vascular/renal capsular/perinephric fat invasion (pT1a, Nx, Mx) at the inferior pole and papillary noninvasive carcinoma (pTa, Nx, Mx) at the superior pole. 

Patient did well postoperatively and was discharged for postoperative rehabilitation. She is being followed up regularly as an out-patient showing improvement in appetite and energy levels.

## 3. Discussion

Renal cell carcinoma represents 3% of adult cancers, with 20% invading the collecting system or capsule [1]. Ten percent of renal tumors arise in the renal pelvis, of which 90% are UC [2]. The documented occurrence of both of these types of tumors in the same kidney is extremely rare, with the literature limited to a few small series and case reports. In the English language literature, the first report was by Graves and Templeton in 1921 and the most recent by Demir and colleagues in 2004 and Lee et al. in 1994, respectively [3–5]. A Spanish language review and case series reported 47 described cases and suggested no worse prognosis associated with this dual pathology [6]. 

The prognosis for a patient with dual malignancies is likely most influenced by the more aggressive of the two tumors. Our patient's RCC was pathologically a pT1a lesion with no evidence of metastatic disease. Our patient's UC was pathologically high grade and a pT3 lesion, with involvement of renal sinus fat via invasion through muscle. A renal pelvic location has improved survival in UC versus a ureteral primary [7, 8], though Holmang and Johansson [9] have suggested only 25% survival in high-grade pT3 upper tract UC. The UC was likely the more ominous primary lesion in this patient. 

Isolated cases of synchronous ipsilateral renal malignancies presenting as pyelonephritis have been reported [10].

The patient had an uneventful in-hospital course and was well at her first follow-up clinic visit after surgery. Further followup will combine aspects of recommended followup for both RCC and UC, as per published guidelines [11]. The lessons learned in this case are to be aware of the possibility of synchronous renal tumours, as they do rarely occur, and to request intraoperative pathology consultation in the setting of nephrectomy for unusual renal lesions. In medicine, usually one explanation suffices, in this case putatively RCC with invasion into the renal pelvis. This diagnosis might have explained the hematuria, negative cytology, and large mass consistent with RCC on CT scan. In this case, 2 cancers presented together in a typical manner, but their simultaneous presence was definitely unexpected. 

## Figures and Tables

**Figure 1:**
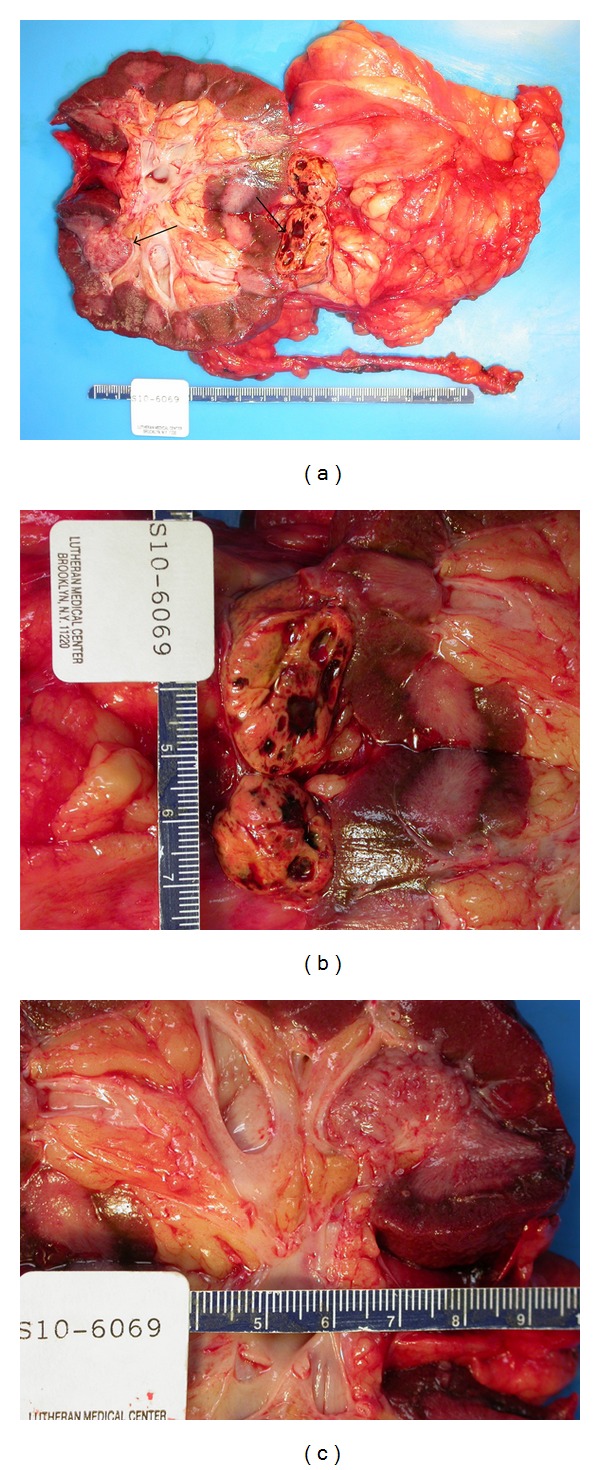
Right kidney nephrectomy specimen (a). A thin arrow denotes the renal cell carcinoma (b); thick arrow designates the separate synchronous low-grade papillary urothelial carcinoma (c).

**Figure 2:**
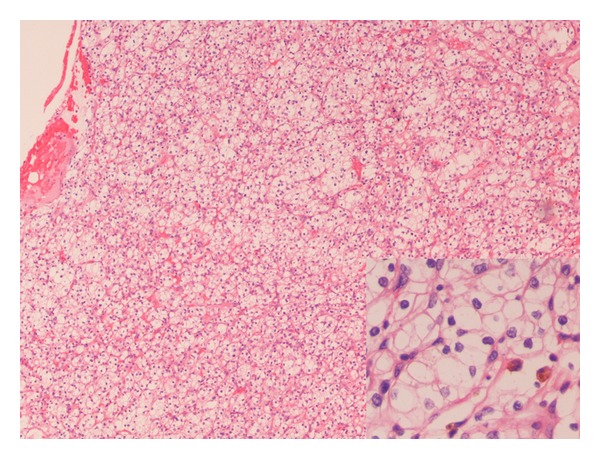
Section of the tumor at the inferior pole of the right kidney, showing the classic features of renal cell carcinoma, clear cell type (low power, 10x objective). The cytoplasm is optically clear, and the nuclei are slightly irregular with inconspicuous nucleoli only visible at high power (40x objective, inset) consistent with Fuhrman nuclear grade 2 of 4.

**Figure 3:**
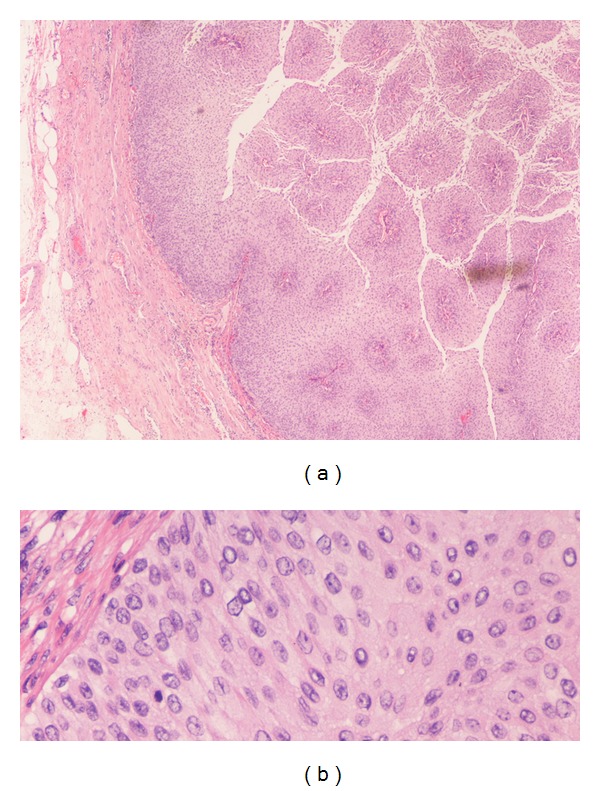
Section of the tumor at the superior renal pelvis, showing low-grade papillary transitional cell carcinoma, consisting of branching papillary fronds with fibrovascular cores ((a) low-power 4x objective). Note that tumor is confined to epithelium, with no invasion into the lamina propria or beyond. High-power view ((b) 40x objective) shows an orderly appearance of neoplastic cells with only mild nuclear pleomorphism and inconspicuous nucleoli.
